# Prognostic Role of ceRNA Network in Immune Infiltration of Hepatocellular Carcinoma

**DOI:** 10.3389/fgene.2021.739975

**Published:** 2021-09-13

**Authors:** Qianhui Xu, Shaohuai Chen, Yuanbo Hu, Wen Huang

**Affiliations:** The Second Affiliated Hospital and Yuying Children’s Hospital of Wenzhou Medical University, Wenzhou, China

**Keywords:** hepatocellular carcinoma, ceRNAs, prognostic prediction, tumor immune microenvironment, immunotherapy, tumor mutation burden

## Abstract

**Background:** Increasing evidence supports that competing endogenous RNAs (ceRNAs) and tumor immune infiltration act as pivotal players in tumor progression of hepatocellular carcinoma (HCC). Nonetheless, comprehensive analysis focusing on ceRNAs and immune infiltration in HCC is lacking.

**Methods:** RNA and miRNA sequencing information, corresponding clinical annotation, and mutation data of HCC downloaded from The Cancer Genome Atlas Liver Hepatocellular Carcinoma (TCGA-LIHC) project were employed to identify significant differentially expressed mRNAs (DEMs), miRNAs (DEMis), and lncRNAs (DELs) to establish a ceRNA regulatory network. The Kyoto Encyclopedia of Genes and Genomes (KEGG) and Gene ontology (GO) enrichment pathways were analyzed to functionally annotate these DEMs. A multigene-based risk signature was developed utilizing least absolute shrinkage and selection operator method (LASSO) algorithm. Moreover, survival analysis and receiver operating characteristic (ROC) analysis were applied for prognostic value validation. Seven algorithms (TIMER, XCELL, MCPcounter, QUANTISEQ, CIBERSORT, EPIC, and CIBERSORT-ABS) were utilized to characterize tumor immune microenvironment (TIME). Finally, the mutation data were analyzed by employing “maftools” package.

**Results:** In total, 136 DELs, 128 DEMis, and 2,028 DEMs were recognized in HCC. A specific lncRNA–miRNA–mRNA network consisting of 3 lncRNAs, 12 miRNAs, and 21 mRNAs was established. A ceRNA-based prognostic signature was established to classify samples into two risk subgroups, which presented excellent prognostic performance. In additional, prognostic risk-clinical nomogram was delineated to assess risk of individual sample quantitatively. Besides, risk score was significantly associated with contexture of TIME and immunotherapeutic targets. Finally, potential interaction between risk score with tumor mutation burden (TMB) was revealed.

**Conclusion:** In this work, comprehensive analyses of ceRNAs coexpression network will facilitate prognostic prediction, delineate complexity of TIME, and contribute insight into precision therapy for HCC.

## Introduction

Primary liver cancer is considered as one of the most aggressive and prevalent malignancies with increasing mortality globally (Bray et al., 2018; Forner et al., 2018; Yang et al., 2019). Based on conventional histopathological classification, hepatocellular carcinoma (HCC) almost take part in 75–85% of primary liver cancer patients (Bray et al., 2018). Such underlying pathogenic elements for HCC such as infections of aflatoxin exposure, hepatitis virus, heavy alcohol intake, type 2 diabetes, and obesity served as crucial players in hepatocarcinogenesis (Yang et al., 2019; Xu et al., 2021c). Because of complex molecular diversity like genetic and genomic alternation, HCC is a highly heterogeneous solid tumor both in terms of intertumor and intratumor standpoint (Schulze et al., 2016; Cancer Genome Atlas Research Network, 2017; Liu et al., 2018; Woo and Kim, 2018). Since HCC has considerably high heterogeneity and sophisticated diversity of etiology, tumor–node–metastasis (TNM) staging has been difficult in the precise prognostic prediction of HCC patients (Edge and Compton, 2010; Marano et al., 2015). It is of great urgency, therefore, to construct a novel and reliable predictive indicator for clinical outcome prediction and therapeutic efficacy estimation, further advancing tailored strategy.

The recently improved therapeutic efficacy by immune checkpoint blockade (ICB) therapy, eliciting anticancer effect by blocking cytotoxic T-lymphocyte-associated antigen 4 (CTLA-4) and programmed cell death protein 1 and its ligand (PD-1/PD-L1), has made a breakthrough in malignant cancers (Brahmer et al., 2015; Weber et al., 2015; Cella et al., 2016; Reck et al., 2018). Clinical trials presented that almost 31% of HCC cases experienced durable benefits from immunotherapy, suggesting their encouraging potential in lifesaving (El-Khoueiry et al., 2017; Xu et al., 2021b). Accumulating evidence supported that coordination of the immunological regulators acts as a pivotal role in cancer development and sensitivity to treatment (Nishida and Kudo, 2017). An independent study reported that exhaustion of CD4 + T cells resulted in acceleration of HCC (Ma et al., 2016; Xu et al., 2021a). Lymphotoxin-α and lymphotoxin-β produced by CD8 + T cells may serve as key promoters in the progression of HCC (Finkin et al., 2015). Tumor mutation burden (TMB), representing the somatic coding errors such as base substitutions, deletions across, or insertions per million bases, has been considered as an encouraging predictive factor for immunotherapeutic effect prediction (Snyder et al., 2014; Rizvi et al., 2015; Chan et al., 2019). High TMB was discovered to promote antigen formation followed by immune cells infiltration, resulting in enhanced immunotherapeutic effect (Miao et al., 2018). Currently, a large number of researches have highlighted the association of immunotherapy and TMB in HCC (Hellmann et al., 2018; Chan et al., 2019). However, there is little knowledge on the correlation of TMB with competing endogenous RNA (ceRNA) in HCC.

The hypothesis of ceRNA proposed a novel mechanism for interactions of non-coding RNA and mRNA, in which long non-coding RNAs (lncRNAs), microRNAs (miRNAs), and mRNA messenger RNAs (mRNAs) participated (Salmena et al., 2011). In the regulatory ceRNA networks, miRNA is able to bind to the 3′ untranslated regions (3′UTRs) of RNAs to inhibit the translation of the target genes (Grimson et al., 2007). lncRNAs can competitively bind miRNAs by sharing miRNA response elements with reverse complementary binding seed regions to indirectly affect translational regulation and mRNA stability (Cao et al., 2018). Accumulating researches highlighted that the ceRNA gene interaction network served a critical role in HCC tumorigenesis, progression, and prognosis (Jeyaram et al., 2018). For example, HULC participated in the pathogenesis of HCC by combining with miR-372 to influence gene expression of PRKACB (Wang et al., 2010). Another research reported that the lncRNA DSCR8 activated frizzled-7 by competitively sponging the miRNA miR-485-5p to promote tumor progression (Wang et al., 2018). However, the underlying mechanism of ceRNA regulatory networks in prognostic prediction, tumor immune infiltration, immunotherapy, and TMB estimation of HCC remains elusive.

In this work, normal and tumor samples of HCC were obtained from The Cancer Genome Atlas (TCGA) database; the DESeq2 method was employed to determine differentially expressed mRNAs (DEMs), miRNAs (DEMis), and lncRNAs (DELs) between tumor tissues and normal tissues. Then, Kyoto Encyclopedia of Genes and Genomes (KEGG) and Gene ontology (GO) pathway enrichment analysis were explored to further predict potential biological functions and activating signaling pathways. In total, 26 mRNAs, 10 miRNAs, and 37 lncRNAs were identified to establish regulatory ceRNA network specific to HCC. After least absolute shrinkage and selection operator method (LASSO)-penalized Cox regression analysis, seven ceRNAs with significant prognostic value were identified to construct a prognostic signature. Kaplan–Meier (K-M) survival analysis and receiver operating characteristic (ROC) analysis were applied for prognostic value validation. Besides, prognostic nomogram was constructed to quantitatively measure risk, and risk score was significantly associated with diversity of tumor immune microenvironment (TIME). Finally, intrinsic link between risk score with TMB was explored. Herein, comprehensive analyses of ceRNA network will facilitate prognostic prediction, delineate complexity of TIME, and contribute insight into precision immunotherapy for HCC.

## Materials and Methods

### Data Collection and Differential Gene Expression Analysis

The RNA and miRNA sequence data of the normal liver tissues and HCC samples were obtained from TCGA database. All file data were downloaded using the GDC Data Transfer Tool. The demographic information (age, gender, and so on), survival endpoint (vital status, days to last follow-up and days to death), and clinical stage and grade of tumor of each sample were also downloaded.

Next, four categories of somatic mutation data of HCC patients were obtained from TCGA portal. We singled out the mutation files, which were obtained through the “SomaticSniper variant aggregation and masking” platform for subsequent analysis.

### Identification of Differentially Expressed Genes (DEGs)

Then, the DESeq2 method with an adjusted *p*-value < 0.05 and | log2 fold change (FC)| > 1 setting as the threshold was employed to identify differentially expressed mRNAs (DEmRNAs), miRNAs (DEmiRNAs), and lncRNAs (DElncRNAs) between normal and tumor samples. Taking advantage of pheatmap R package (Version: 1.0.12), a hierarchical cluster heatmap representing the expression direction and intensity of DEGs was plotted.

### Construction of the ceRNA Network

Firstly, interactions between lncRNA and miRNAs were predicted using the miRcode database^1^ (Jeggari et al., 2012). After that, the TargetScan, mirTarBase, and miRDB (Hsu et al., 2011; Wong and Wang, 2015) databases were used to retrieve the miRNA–mRNA interaction. Finally, a ceRNA network was visualized using Cytoscape v.3.8.0.

### Functional Enrichment Analysis

The Entrez ID for each DEmRNA was obtained using R package “org.Hs.eg.db.” To elucidate underlying mechanisms of the hub genes related to DEmRNA in the biological process (BP), GO and KEGG function annotations were analyzed with “ggplot2,” “enrichplot,” and “clusterProfiler” packages.

### Construction of the Risk Score System

All components of the ceRNA network associated with prognosis were analyzed through LASSO-penalized Cox regression to assure multifaceted models were not overfitting. Cox proportional hazards model was established using the penalized maximum likelihood algorithm. Ten-fold cross-validation was utilized to derive the best lambda to minimize the mean cross-validated error and predict the regression coefficients (β) of the multivariate Cox regression model. Then, a prognostic model including seven genes was developed, and risk score was calculated with the formula below. Risk score = βgene 1 × expression level of gene 1 + βgene 2 × expression level of gene 2 + ⋯ + βgene n × expression level of gene n. Herein, β was the regression coefficient in the multivariate Cox regression analysis as described previously (Lossos et al., 2004).

### Validation of the Prognostic ceRNA-Based Signature

According to the previous risk formula, each HCC sample obtained corresponding risk score. All samples were stratified into high- and low-risk clusters when setting the median risk scores as the cutoff point. To visualize the correlation of risk score with clinicopathological variables, R “pheatmap” package was employed and the clinical characteristics between low- and high-risk patients were compared. Next, K-M survival curve was plotted using R package “survival” to identify prognosis difference. Moreover, time-dependent ROC curves were analyzed to validate prognosis predictive performance. Then, univariate and multivariate Cox regression analyses were performed for validity of risk signature as an independent prognostic indicator.

### Establishment and Verification of the Nomogram

To identify the optimal prognostic indicator, risk score, age, gender, tumor grade, and clinicopathological stage for 1/2/3-year overall survival (OS), ROC analysis was performed (Blanche et al., 2013). To develop a quantitative prognostic pool for HCC patients, a nomogram plot integrating risk score and other clinicopathological features was constructed to predict 1–, 2–, and 3-year OS rate. Then, the calibration curve, which could present predictive validity of the nomogram, was plotted.

### Risk Score in Characterization of TIME

To elucidate the potential role of risk score in TIME contexture, seven methods including TIMER, XCELL, MCPcounter, QUANTISEQ, CIBERSORT, CIBERSORT-ABS, and EPIC were employed to estimate immune infiltration. Spearman correlation analysis was performed to investigate the association of risk score with TIME characterization.

### Prediction of Response to Immunotherapy of the Patients

Based on published studies, expression levels of ICB-related genes exhibited intimate interaction with immunotherapeutic efficacy (Goodman et al., 2017). Herein, six ICB-related genes were extracted: PD-1 (also known as PDCD1), PD-L1 (also known as CD274), PD-L2 (also known as PDCD1LG2), T-cell immunoglobulin domain and mucin domain-containing molecule-3 (TIM-3, also known as HAVCR2), indoleamine 2,3-dioxygenase 1 (IDO1), and cytotoxic T-lymphocyte antigen 4 (CTLA-4) (Kim et al., 2017; Nishino et al., 2017; Zhai et al., 2018). To reveal the underlying players of risk score in immunotherapy, correlation analysis of risk score with these ICB-related genes expression levels was performed.

### Collection and Preprocess of Epigenetic Mutation Data

The corresponding somatic alteration information of The Cancer Genome Atlas Liver Hepatocellular Carcinoma (TCGA-LIHC) cohort was obtained from the TCGA dataset. TMB was defined as the number of somatic, coding, base replacement, and insert–deletion mutations per megabase of the genome examined using non-synonymous and code-shifting indels under a 5% detection limit. The “maftools” R package (Mayakonda et al., 2018) was employed to detect the number of somatic non-synonymous point mutations within each sample.

### Statistical Analysis

The Wilcoxon test was employed to compare two groups, whereas the Kruskal–Wallis test was carried out to compare more than two groups. Survival curves were analyzed by the K-M log rank test. The chi-square test was performed to correlate the risk score subgroups with somatic mutation frequency, and the Spearman analysis computed the correlation coefficient. Results of CIBERSORT algorithm with *p* < 0.05 were adopted in the subsequent analysis. Two-tailed *p* < 0.05 deemed statistical significance. R software (version 4.0.3) was used for all statistical analyses.

## Results

### Identification of Different Expressed Genes

**FIGURE 1:**
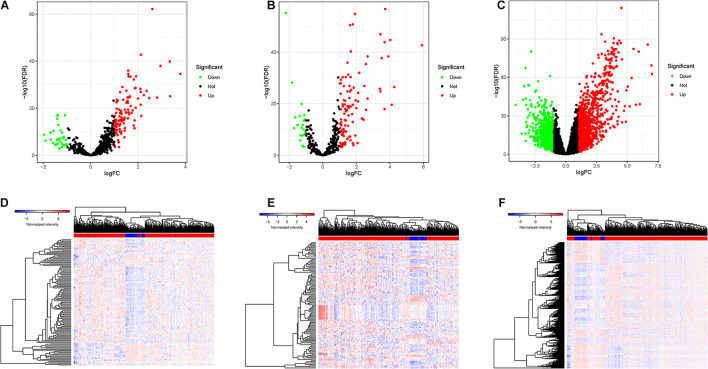
The DEGs between HCC samples and normal liver tissues. **(A)** Volcano plots of the expression levels of DELs. **(B)** Heatmaps of the expression levels of DELs. Red represents upregulated expression, and green represents downregulated expression. **(C)** Volcano plots of the expression levels of DEMis. **(D)** Heatmaps of the expression levels of DEMis. **(E)** Volcano plots of the expression levels of DEMs. **(F)** Heatmaps of the expression levels of DEMs.

DElncRNAs, DEmiRNAs, and DEmRNAs were analyzed between 374 HCC tissues and 50 adjacent normal liver samples in the TCGA database. After setting an adjusted *p*-value < 0.05 and | log2 FC| > 1 as cutoff threshold, a total of 136 lncRNAs (104 upregulated and 32 downregulated; Figure 1A), 128 miRNAs (107 upregulated and 21 downregulated; Figure 1C), and 2,028 protein-coding genes (1,222 upregulated and 806 downregulated; Figure 1E) were differently expressed between HCC samples and normal tissues. Clustering analysis of DElncRNAs, DEmiRNAs, and DEmRNAs suggested that HCC samples may be distinguished from normal samples according to expression profiling of DEmRNAs, DEmiRNAs, and DElncRNAs (Figures 1B,D,F). The detailed information of DElncRNAs, DEmiRNAs, and DEmRNAs are listed in Supplementary Table 1.

### Construction of ceRNA Regulatory Network and Enrichment Analysis

**FIGURE 2:**
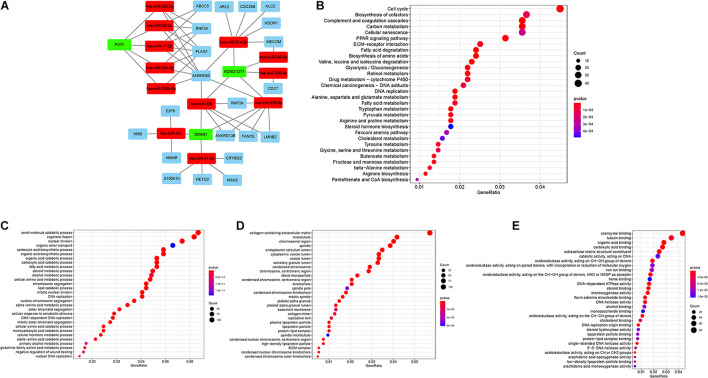
**(A)** lncRNA–miRNA–mRNA ceRNA network in HCC. Green, red, and blue represent lncRNAs, miRNAs, and mRNAs, respectively. **(B)** KEGG enrichment analysis of DEMs. **(C–E)** GO enrichment analysis of DEMs: BPs, CCs, and MF.

The interactions among DEmRNA, DElncRNA, and DEmiRNAs were predicted using different databases to construct the ceRNA regulatory network. A ceRNA regulatory network was established including 36 genes (3 lncRNAs, 12 miRNAs, and 21 mRNAs). All these genes made up the interactions of 14 lncRNA–miRNA pairs and 31 miRNA–mRNA pairs (Figure 2A and Supplementary Table 1). To explore the potential role of DEmRNAs in physiological process, GO and KEGG pathway enrichment were analyzed (Supplementary Tables 3, 4). For KEGG analysis, the top enriched terms were Cell cycle, Biosynthesis of cofactors, and Complement and coagulation cascades (Figure 2B). The result of the GO enrichment pathways presented that the DEmRNAs were primarily enriched in small-molecule catabolic process, organelle fission, and nuclear division in BPs; chromosomal region, microtubule, and collagen-containing extracellular matrix in cellular components (CCs); and coenzyme binding, tubulin binding, and organic acid binding in molecular function (MF; Figures 2C–E).

### Construction of Prognostic Risk Signature

**FIGURE 3:**
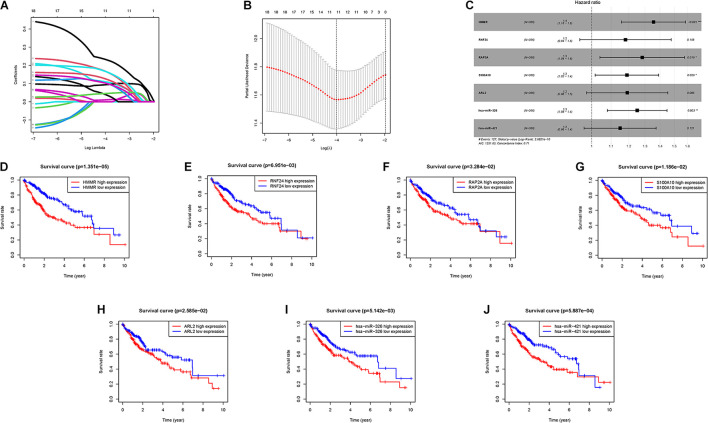
Establishment of the prognostic risk signature. **(A)** LASSO coefficient profiles of 36 candidate genes. A vertical line is drawn at the value chosen by 10-fold cross-validation. **(B)** Ten-time cross-validation for tuning parameter selection in the LASSO regression. The vertical lines are plotted based on the optimal data according to the minimum criteria and 1-standard error criterion. The left vertical line represents the 11 genes finally identified. **(C)** Forest plots showing the relationships of each gene subsets with OS. The unadjusted HRs are presented with 95% CIs. **(D)** K-M curve analysis presenting difference of OS between the high-HMMR and low-HMMR groups. **(E)** K-M curve analysis presenting difference of OS between the high-RNF24 and low-RNF24 groups. **(F)** K-M curve analysis presenting difference of OS between the high-RAP2A and low-RAP2A groups. **(G)** K-M curve analysis presenting difference of OS between the high-S100A10 and low-S100A10 groups. **(H)** K-M curve analysis presenting difference of OS between the high-ARL2 and low-ARL2 groups. **(I)** K-M curve analysis presenting difference of OS between the high-hsa-miR-326 and low-hsa-miR-326 groups. **(J)** K-M curve analysis presenting difference of OS between the high-hsa-miR-421 and low-hsa-miR-421 groups.

With the help of univariate Cox analysis, 19 ceRNA genes were identified with significant prognostic value (*p* < 0.05, Supplementary Table 5). In order to avoid overfitting the risk score model, LASSO Cox regression was conducted on the abovementioned hub genes and then recognized 11 ceRNA genes associated with prognosis in HCC (Figure 3A), and the optimal values of the penalty parameter were determined by 10-round cross-validation (Figure 3B). Next, multivariate Cox regression was performed, seven ceRNA genes (RNF24, HMMR, RAP2A, ARL2, S100A10, hsa-miR-421, and hsa-miR-326) were determined as the hub genes, all of which were considered as unfavorable prognostic indicator (all HRs > 1; Figure 3C, Supplementary Table 6). Furthermore, survival analysis showed that abnormal mRNA expression of most hub genes resulted in significant different OS times between low- and high-gene-expression subgroups (all *p* < 0.05; Figures 3D–J).

Subsequently, these seven hub genes were incorporated into a risk score model, and risk score was computed as follows: risk score = (0.3039 ^∗^ expression value of HMMR) + (0.1663 ^∗^ expression value of RNF24) + (0.2488 ^∗^ expression value of RAP2A) + (0.1732 ^∗^ expression value of S100A10) + (0.175 ^∗^ expression value of ARL2) + (0.224 ^∗^ expression value of hsa-miR-326) + (0.1399 ^∗^ expression value of hsa-miR-421). Finally, each HCC sample with corresponding risk score was clustered into high-/low-risk subgroups.

### Validation of Risk Prognostic Signature

**FIGURE 4:**
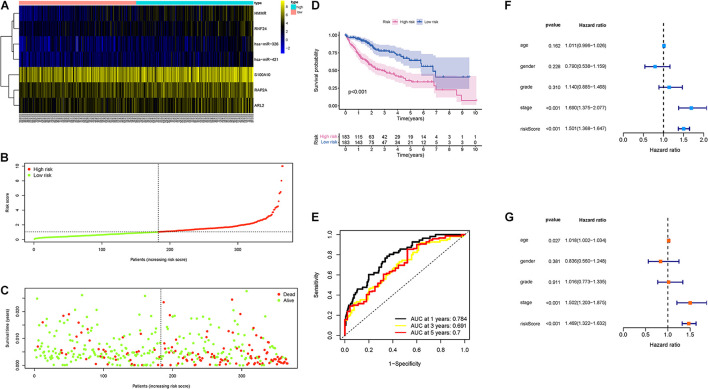
Validation of the prognostic value of risk signature. **(A)** Heatmap presents the expression pattern of three hub genes in each sample, where the colors of yellow to blue represented alterations from high expression to low expression. **(B)** Distribution of multigene signature risk score. **(C)** The survival status and interval of HCC patients. **(D)** K-M curve analysis presenting difference of OS between the high-risk and low-risk groups. **(E)** Areas under the curve (AUCs) of the risk scores for predicting 1–, 2–, and 3-year OS time. **(F)** Univariate Cox regression analyses of OS. **(G)** Multivariate Cox regression analyses of OS.

First, the distributions of these seven genes with corresponding groups and samples were delineated in Figure 4A. The distributions of dot pot and risk score of survival status suggested that low-risk patients had longer OS time (Figures 4B,C). Besides, K-M survival curve demonstrated that high-risk samples presented significantly shorter OS time than patients with low-risk (*p* < 0.001; Figure 4D). Additionally, ROC curves were plotted, and AUC values for the 1–, 2–, and 3-year OS reached 0.784, 0.691, and 0.7, respectively (Figure 4E). Then, univariate Cox analysis pointed out that the hazard ratio (HR) of the risk score was 1.501 (95% confidence interval (CI): 1.368-1.647; Figure 4F). The results of the multivariate Cox proportional hazards model (HR = 1.469, 95% CI: 1.322–1.632; Figure 4G) supported the risk score performed as an independent prognostic indicator in HCC. These results suggested an excellent capacity of our multigene signature for clinical outcome prediction.

### Risk Score in Clinical Features

**FIGURE 5:**
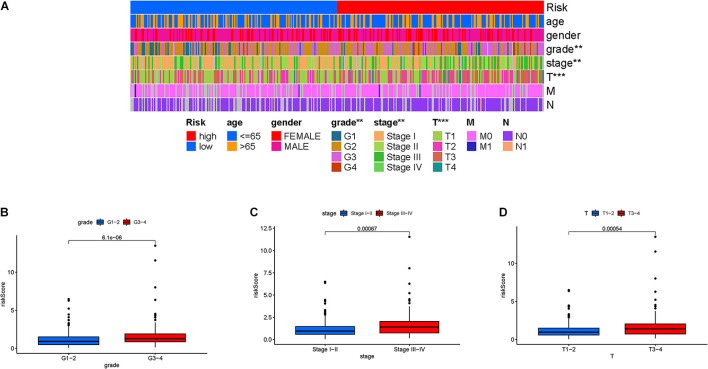
Clinical significance of the prognostic risk signature. **(A)** Heatmap presents the distribution of clinical feature and corresponding risk score in each sample. Comparison of risk score among different subgroups classified by clinical characteristics: **(B)** WHO grade, **(C)** clinical stage, and **(D)** T status.

Firstly, the distribution of clinical variables with corresponding risk subgroups was visualized (Figure 5A). For early-grade samples and late-grade samples, risk score presented a higher trend in late-grade samples (Figure 5B). We also observed that patients with advanced stage also exhibited a significant increase in risk score (Figure 5C). Similarly, risk score was significantly elevated in T3-4 status (Figure 5D).

Stratification analysis was employed to validate whether risk score still could identify difference of prognosis when HCC patients were subgrouped into clinical variable groups. When patients were divided based on age, we found that our risk score was still predictive of patient outcomes, with higher scores indicating poorer outcomes (Supplementary Figures 2A,B). Likewise, risk score presented powerful prognostic ability for male or female patients (Supplementary Figures 2C,D), 1–2 or 3–4 clinical grade patients (Supplementary Figures 2E,F), patients in early and late stage (Supplementary Figures 2G,H), patients in T1-2 or T3-4 status (Supplementary Figures 2I,J), patients in N0 category (Supplementary Figure 2K), and patients in M0 category (Supplementary Figure 2L). The above findings, combined with the results of univariable and multivariable regression analyses, emphasized that our risk score was indeed an outstanding prognostic predictor independent from other clinical parameters.

### Construction of Prognostic Nomogram

**FIGURE 6:**
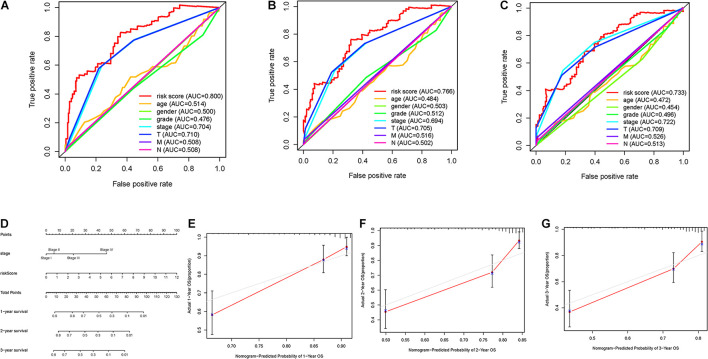
Validation of prognostic efficiency of risk signature. **(A–C)** AUCs of the risk scores for predicting 1–, 2–, and 3-year OS time with other clinical characteristics. **(D)** Nomogram was assembled by stage and risk signature for predicting survival of HCC patients. **(E)** One-year nomogram calibration curves. **(F)** Two-year nomogram calibration curves. **(G)** Three-year nomogram calibration curves.

To further investigate whether risk score was the best predictive factor among multiple clinicopathological variables, age, gender, clinical staging, tumor grade, T status, and N status were listed as candidate prognostic indicators. These clinical variables were incorporated to perform the AUC analysis for 1–, 2–, and 3-year OS, and risk score exhibited the most AUC value (Figures 6A–C). Then, a prognostic nomogram consisting of clinical stage and risk score was developed for quantitative prognosis prediction (Figure 6D). Gender, stage, grade, T status, and N status were excluded out of nomogram given, of which AUC values were less than 0.6. In addition, calibrate curves suggested excellent prognosis predictive performance of the nomogram model (Figures 6E–G).

### Correlation of Risk Signature With Immune Cells Infiltration

**FIGURE 7:**
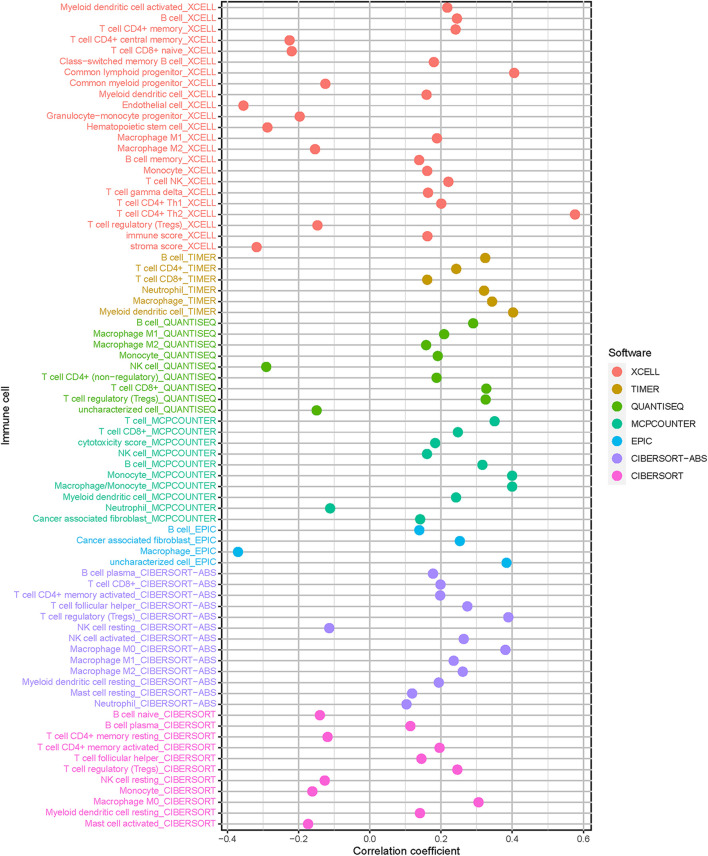
Estimation of tumor-infiltrating cells. Patients in the high-risk group were more positively associated with cancer associated fibroblast, M2 macrophage, and T cell regulatory, whereas they were negatively associated with resting NK cells and resting memory CD4 + T cells, as shown by Spearman correlation analysis.

Since HCC progression and infiltration immune cells had intrinsic and intimate connection, we further explored the potential contribution of risk signature in diversity and complexity of TIME. The result presented that risk score was remarkably and negatively correlated with populations of resting NK cells and resting memory CD4 + T cells, whereas positively related with infiltration of cancer associated fibroblast, M2 macrophage, and T regulatory cells (Tregs; Supplementary Figures 3–6). Furthermore, Spearman correlation of risk score with immune infiltration was further analyzed (Figure 7), and the detailed results are provided in Supplementary Table 7.

### Predicting Clinical Outcome of Patients to Immunotherapy

**FIGURE 8:**
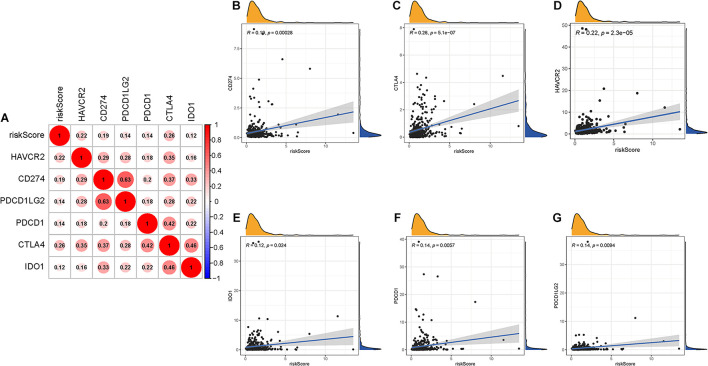
Correlation between prognostic risk signature with hub immune checkpoint genes. **(A)** Correlation analysis between immune checkpoint inhibitors (CD274, PDCD1, PDCD1LG2, CTLA4, HAVCR2, and IDO1) with prognostic risk signature. **(B)** Correlation between prognostic risk signature and CD274. **(C)** Correlation between prognostic risk signature and CTLA4. **(D)** Correlation between prognostic risk signature and HAVCR2. **(E)** Correlation between prognostic risk signature and IDO1. **(F)** Correlation between prognostic risk signature and PDCD1. **(G)** Correlation between prognostic risk signature and PDCD1LG2.

Given that the information on immunotherapy was not available in the TCGA-LIHC dataset, further analysis was explored for response to immunotherapy. Firstly, correlation of ICB-related gene (CD274, PDCD1, PDCD1LG2, IDO1, HAVCR2, and CTLA-4) (Kim et al., 2017; Nishino et al., 2017; Zhai et al., 2018) mRNA expression level with risk score was performed (Figure 8A). It was discovered that risk score was significantly and negatively correlated with CD274 (*r* = 0.19; *p* = 0.00028), CTLA4 (*r* = 0.26; *p* = 5.1e–07), HAVCR2 (*r* = 0.22; *p* = 2.3e–05), IDO1 (*r* = 0.12; *p* = 0.024), PDCD1 (*r* = 0.14; *p* = 0.0057), and PDCD1LG2 (*r* = 0.14; *p* = 0.0094; Figures 8B–G), suggesting that patients with high risk score may be more blocked by immune checkpoint administration.

### The Association Between the Risk Signature With TMB

**FIGURE 9:**
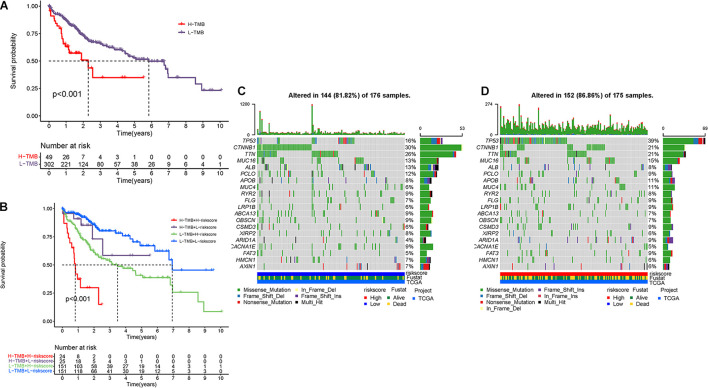
The correlation between the risk score and TMB. **(A)** K-M curves for high and low TMB groups. **(B)** K-M curves for patients stratified by both TMB and risk score. The oncoPrint was constructed using low-risk score **(C)** and high-risk score **(D)**.

Mounting researches have highlighted that tumor burden mutation (TMB) was associated with upregulation of CD8 + T cell infiltration, which could identify cancer cells and then execute antitumor response (Rizvi et al., 2015; McGranahan et al., 2016; Chan et al., 2019). For that, we speculated that TMB might act as a non-negligible prognostic factor of responsiveness to antitumor immunotherapy and aimed to investigate the potential interaction between risk score and TMB to uncover the hereditary variations of risk score subtype. Firstly, the TMB level was detected both in low- and high-risk score subgroups. There was no significant distinction of TMB level between the low-risk score subgroup and the high-risk score subgroup (*p* = 0.73, Supplementary Figure 3A). Then, the patients were assigned into distinct subtypes on the line of the TMB immune set point, as stated before (Cristescu et al., 2018). Survival curve demonstrated that high TMB value significantly suggested shorter OS time (*p* < 0.001, Figure 9A). Subsequent correlation analysis further validated that the TMB was positively but not significantly correlated with the risk score (*R* = 0.027, *p* = 0.61; Supplementary Figure 3B). To further explore the validity of consistent prognostic significance of risk score and TMB, we validated the cooperative effect of two indicators in prognostic prediction of HCC. As demonstrated in stratified survival curve, there was no interference of TMB status with risk score prognostic predictive performance. Risk score subgroups exhibited evident prognosis distinctions in both low and high TMB status subtypes (*p* < 0.001; Figure 9B). In summary, these findings indicated that risk score could act as an independent predictor and hold the potential to evaluate the clinical outcome of antitumor immunological treatment.

Besides, we explored and visualized the distribution of gene mutation in both the high-and low-risk score subtypes. The comprehensive landscape of somatic variants visualized the mutation patterns and clinicopathological features of the top 20 driver genes with the most frequent alteration (Figures 9C,D). These findings might contribute novel insight into the intrinsic connection of ceRNA and somatic variants in HCC.

## Discussion

Hepatocellular carcinoma is the one of most malignant and common tumors globally (Bray et al., 2018; Forner et al., 2018; Yang et al., 2019). Such genetic alternation as alternative splicing, TP53 mutation, regulation of non-coding RNA, and DNA methylation served as critical players in progression of HCC (Cancer Genome Atlas Research Network, 2017; Xu et al., 2017; Kahles et al., 2018; Wong et al., 2018). Increasing studies have highlighted the crucial role of immune infiltration in tumor development, especially HCC (Hinshaw and Shevde, 2019; Lu et al., 2019). Immunotherapy with encouraging clinical success has recently emerged as a promising therapeutic strategy in anticancer administration (Yang, 2015). Despite the growing worldwide enthusiasm, several serious challenges remain for HCC immunotherapy, like only 20% of advanced HCC patients presented objective response to immunotherapy (Fu et al., 2019). Such biomolecules as ICB-related targets were not reliable indicators for precise prognostic prediction. As such, predicting prognosis precisely is critical for therapeutic benefit optimization and clinical outcome improvement (Nishino et al., 2017; Mushtaq et al., 2018; Ng et al., 2020). Mounting evidence has supported the suggestion that such non-coding RNAs as lncRNAs and miRNAs played indispensable roles in transcriptional interference and gene regulation (Guttman and Rinn, 2012). Based on the ceRNA hypothesis, a large number of studies have revealed that lncRNA, miRNA, and mRNA interact with each other as corresponding ceRNA networks during cancer occurrence and progression (Schmitt and Chang, 2016; Zhao et al., 2020). Among them, the potential role of ceRNAs in prognostic prediction and tumor microenvironment of HCC caught our eyes. Nevertheless, there is little to know about them before.

Under this background and hypothesis, lncRNA, miRNA, and mRNA data from HCC samples were obtained from the TCGA-LIHC project. In an initial step, DElncRNAs, DEmiRNAs, and DEmRNAs were recognized and integrated by comparing normal tissues with HCC samples. After predicting the lncRNA–miRNA interactions and miRNA–mRNA interactions based on these DEGs, a ceRNA regulatory network composed of 3 lncRNAs, 12 miRNAs, and 21 mRNAs was established to further explore the underlying molecular mechanism of the ceRNAs. In addition, to investigate the potential role of the DEmRNAs in BPs, KEGG and GO pathway enrichment analyses were performed, from which we discovered that DEmRNAs were mostly enriched in cell cycle, biosynthesis of cofactors, and complement and coagulation cascades.

To further validate prognostic value of these genes, we fetched the sequencing profile and clinical information from the TCGA-LIHC project. Subsequently, we conducted univariate, LASSO, and multivariate Cox analyses to identify seven hub genes, and then computed risk score and constructed prognostic signature. The excellent prognostic performance of the risk model was validated by K-M analysis and ROC curves. We demonstrated that risk signature performed well as an independent prognostic predictor by using both univariable and multivariable regression analyses. Additionally, risk signature retained powerful prognostic predictive ability in stratified survival curves based on clinical variables. These results suggested that our seven-gene risk signature could be used as an independent prognostic predictor in HCC. Additionally, prognostic nomogram including risk score and stage was developed to facilitate clinical practice.

Given immune infiltration was a crucial driving factor in HCC, we further investigate the underlying players of risk score in TIME features and immunotherapy. These findings showed that risk score was negatively correlated with abundance of resting immune cell (i.e., resting memory CD4 + T cells, etc.), whereas it was positively correlated with immunosuppressive cells (i.e., cancer-associated fibroblast, Tregs, etc.), indicating low-risk score patients were immune resting phenotype, whereas high-risk score represents immunosuppressive tumor microenvironment. Moreover, risk score was positively and significantly related with the immunotherapy-related genes (i.e., PDCD1, etc.), highlighting high-risk patients might present with a better response for immunotherapy, which needed further exploration in the future.

Currently, several clinical data pointed out a correlation between genetic alternations with responsiveness to immunological treatment (Burr et al., 2017; George et al., 2017). We calculated and determined the TMB, which is a predictive indicator of sensitivity to immunological treatment. Subsequent stratified survival curve demonstrated that risks score held prognostic predictive capability, which was independent of TMB, suggesting that TMB and risk score represent different aspects of immunobiology. Besides, risk score together with mutation data revealed the significant distinction of gene variant frequency between the high- and low-risk score group from the level of transcriptome.

In conclusion, we constructed an lncRNA–miRNA–mRNA ceRNA regulatory coexpression network from a multiomics perspective by using comprehensive bioinformatic analysis. Besides, the distinction of ceRNA-based risk score was demonstrated to contribute to clinical outcome prediction, TIME heterogeneity, and immunotherapeutic response difference. Furthermore, we pointed out the synergistic effect of risk score and TMB value in prognostic prediction. Further experimental and clinical validations are required for these findings at different centers and with larger cohort.

## Data Availability Statement

The datasets presented in this study can be found in online repositories. The names of the repository/repositories and accession number(s) can be found in the article/Supplementary Material.

## Author Contributions

WH designed the overall study and revised the manuscript. QX performed public data interpretation and drafted the manuscript. SC supervised the experiments and contributed to the data analysis. YH participated in data collection. All authors read and approved the final manuscript.

## Conflict of Interest

The authors declare that the research was conducted in the absence of any commercial or financial relationships that could be construed as a potential conflict of interest.

## Publisher’s Note

All claims expressed in this article are solely those of the authors and do not necessarily represent those of their affiliated organizations, or those of the publisher, the editors and the reviewers. Any product that may be evaluated in this article, or claim that may be made by its manufacturer, is not guaranteed or endorsed by the publisher.

## Footnotes

1
http://www.mircode.org/index.php

## Abbreviations

AUC: area under the curve
BP: biological processes
CC: cellular components
CD274: Also known as PD-L1
CI: confidence interval
CTLA-4: cytotoxic T-lymphocyte antigen 4
CTLs: cytotoxic T lymphocytes
DCs: dendritic cells
DEL: deletion
DELs: differentially expressed lncRNAs
DEMs: differentially expressed mRNAs
DEMis: differentially expressed miRNAs
FC: fold change
FDR: false discovery rate
GO: Gene ontology
HCC: hepatocellular carcinoma
HR: hazard ratio
ICB: immune checkpoint blockade
IDO1: indoleamine 2,3-dioxygenase 1
KEGG: Kyoto Encyclopedia of Genes and Genomes
K-M: Kaplan-Meier
K-W: Kruskal-Wallis
LASSO: least absolute shrinkage and selection operator
lncRNAs: long non-coding RNAs
MF: molecular function
mRNAs: mRNA messenger RNAs
miRNAs: microRNAs
OS: overall survival
PDCD1: Also known as PD-1
PDCD1LG2: Also known as PD-L2
ROC: receiver operating characteristic
TCGA: The Cancer Genome Atlas
TICs: tumor-infiltrating immune cells
TILs: tumor infiltrating lymphocytes
TIM-3: T-cell immunoglobulin domain and mucin domain-containing molecule-3
TIME: tumor immune microenvironment
TMB: tumor mutation burden
TNM: tumor node metastasis
Tregs: regulatory T cells.
