# Supplementary material for: Prognostic Role of ceRNA Network in Immune Infiltration of Hepatocellular Carcinoma
Source: Front Genet. 2021 Sep 13;12:739975. doi: 10.3389/fgene.2021.739975 (PMC8473911; doi:10.3389/fgene.2021.739975)
Supplement: Supplementary file 1 [file Data_Sheet_1.pdf]

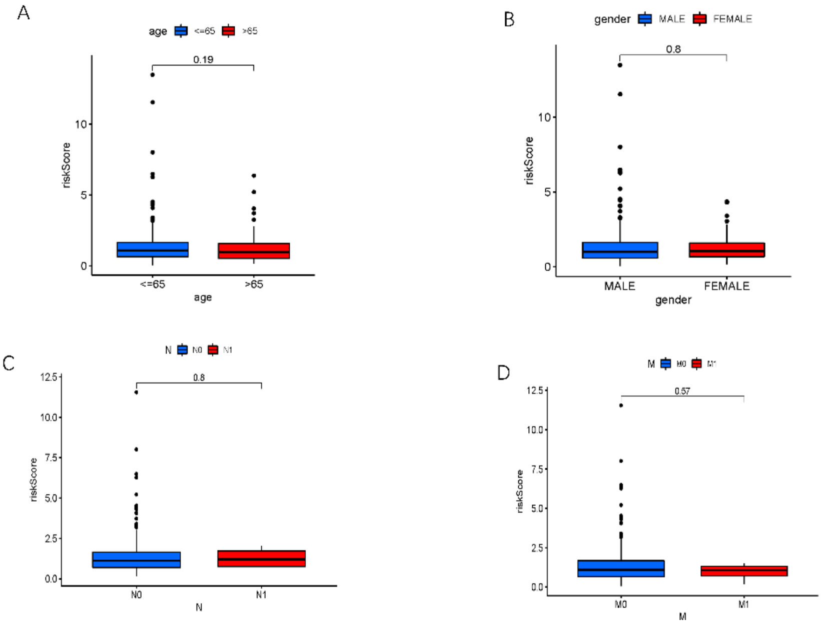

Figure S1: Comparison of risk score among different subgroups classified by clinical characteristics: (A) age, (B) gender, (C) N status, and (D) M status.

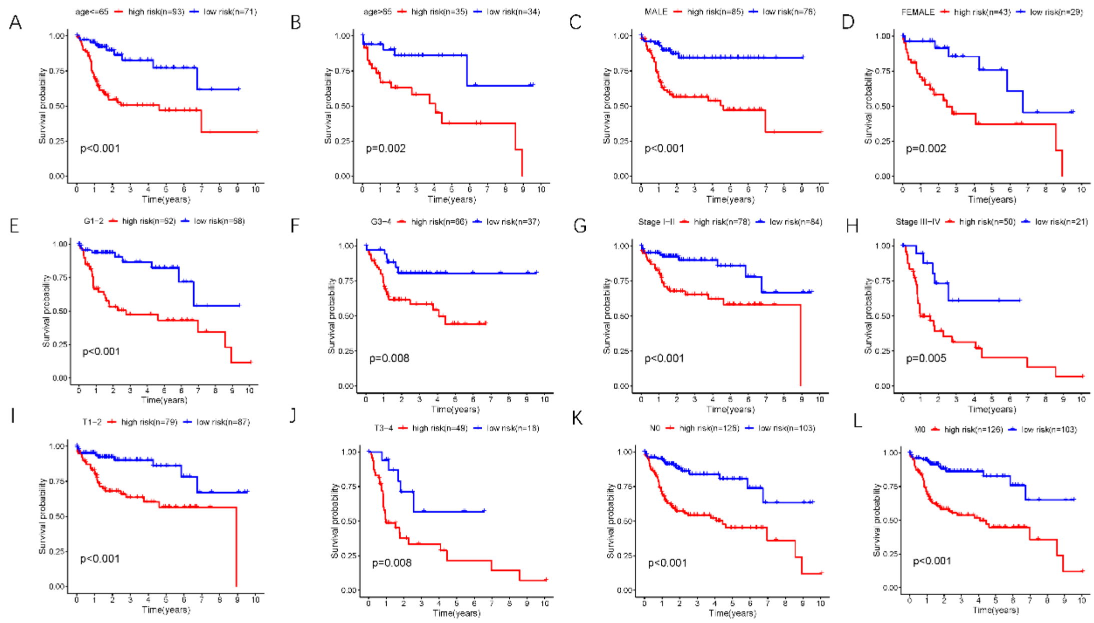

Figure S2: Kaplan–Meier survival analysis for multiple HCC subgroups according to the risk signature stratified by clinical variables. (A-B) Age. (C-D) Gender. (E-F) Tumor grade. (G-H) Stage. (I-J) T status. (K) N status. (L) M status.

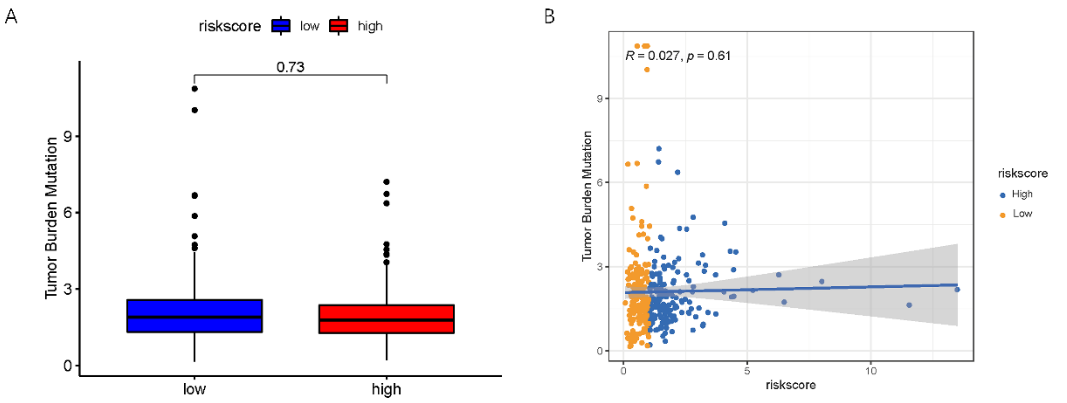

Figure S3: (A) Difference of TMB between patients from the low-/high-risk score subgroups. (B) Scatterplots depicting the positive correlation between risk scores and TMB.

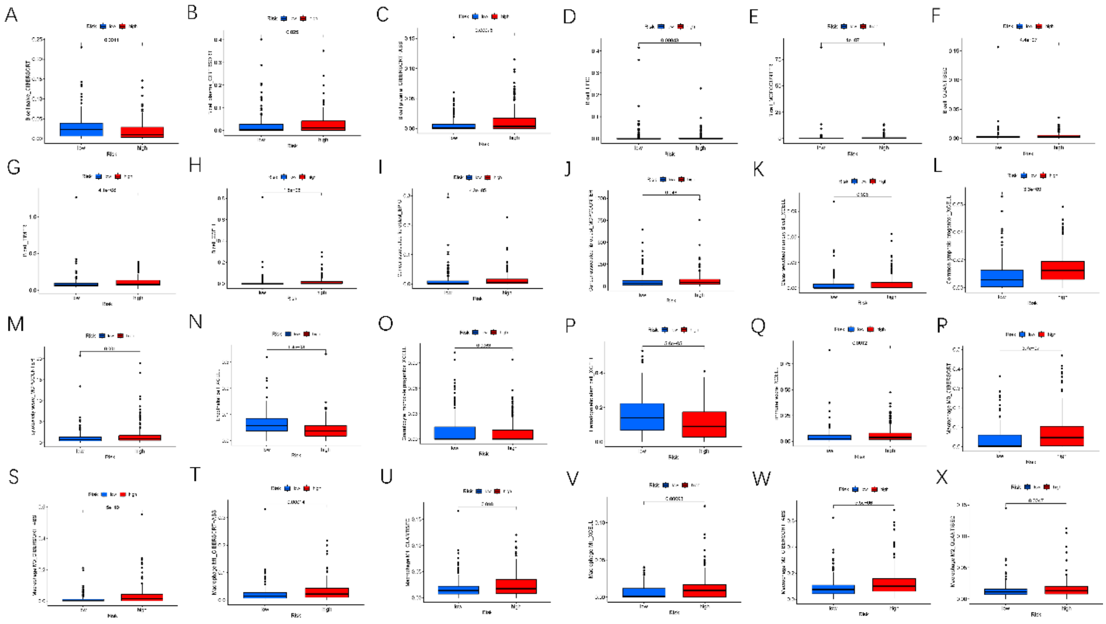

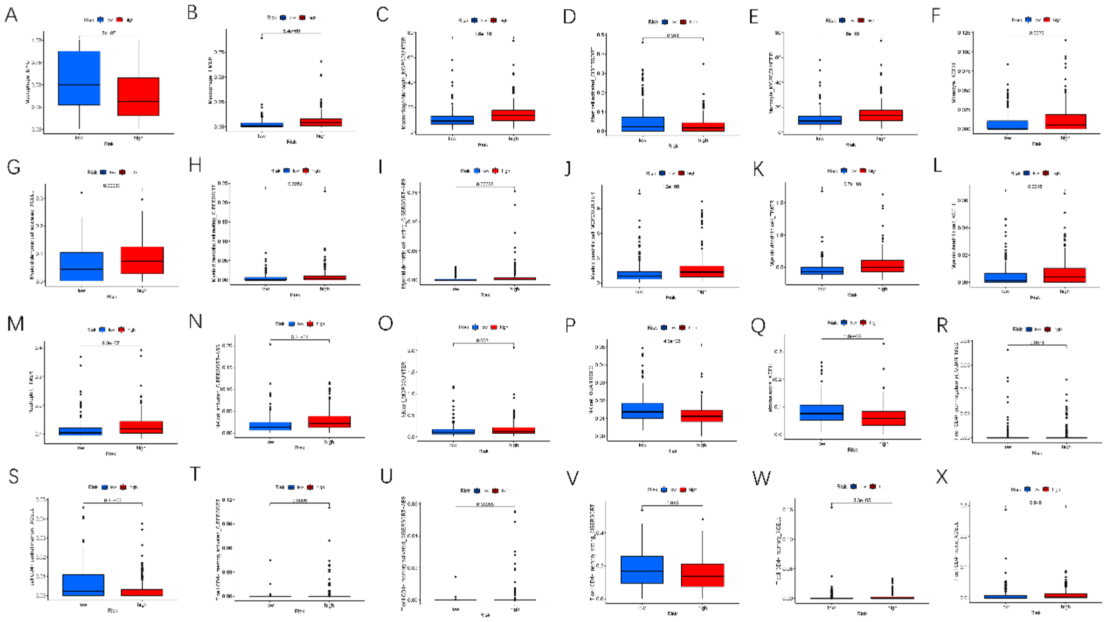

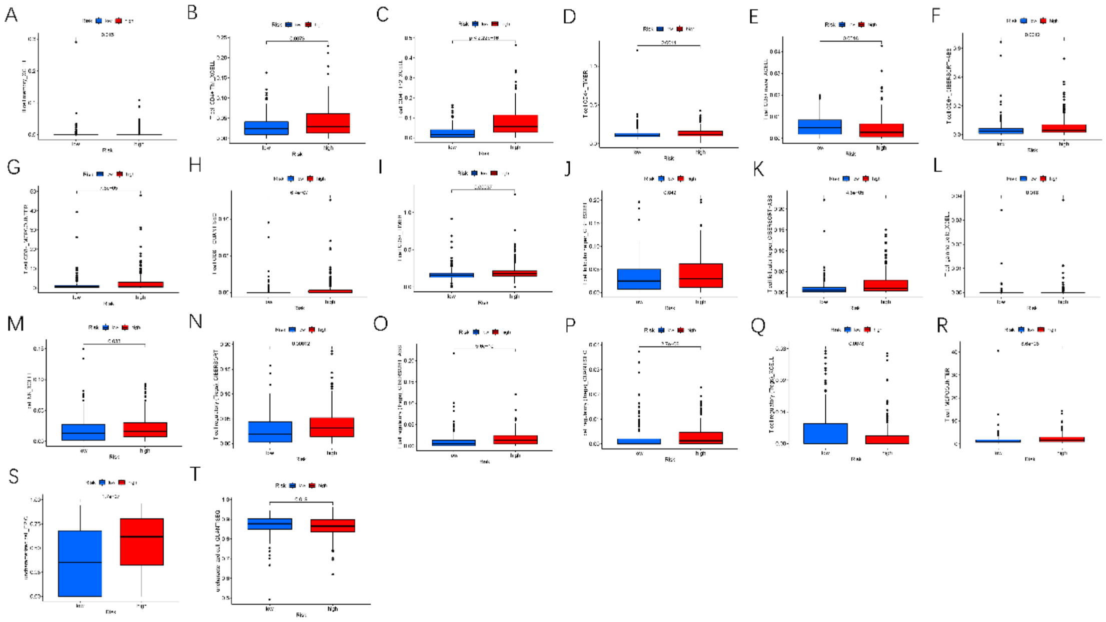

Figure S4-S6: The representative results of the evaluation of tumor infiltrating immune cells with risk signature.
